# Robotic-Assisted Partial Nephrectomy and Adrenalectomy: Case of a Pheochromocytoma Invading into Renal Parenchyma

**DOI:** 10.1155/2020/7321015

**Published:** 2020-06-19

**Authors:** Zeynep G. Gul, Christine W. Liaw, Avinash Reddy, Reza Mehrazin

**Affiliations:** Department of Urology, Icahn School of Medicine at Mount Sinai, New York, NY, USA

## Abstract

Although upper pole renal masses and adrenal masses can usually be distinguished on cross-sectional imaging, large masses can obscure the boundaries between the kidney and adrenal gland. We describe a unique case of an adrenal pheochromocytoma in a 42-year-old female who was referred for robotic partial nephrectomy. During the procedure, the patient developed severe hypertension. The case was aborted, and the workup revealed pheochromocytoma. After appropriate pretreatment, the patient underwent a successful robotic adrenalectomy and partial nephrectomy. Therefore, we recommend screening patients with hypertension and large upper pole masses for pheochromocytoma to better direct preoperative management.

## 1. Introduction

Pheochromocytomas are catecholamine-secreting tumors that arise from the chromaffin cells of the adrenal medulla or the paraganglia. Pheochromocytomas represent about 5% of all adrenal masses [1]. Although they are both located within Gerota's fascia, adrenal and renal masses are usually easily distinguished on imaging. Here, we describe a unique case of an adrenal pheochromocytoma in a 42-year-old African American female who was referred for robotic partial nephrectomy.

## 2. Case Presentation

The patient presented to an outside hospital with chest pain, shortness of breath, and diaphoresis. She was found to be hypertensive and have an elevated troponin with ST depressions on EKG. During the hospital admission, the patient required intubation secondary to hypertensive emergency and flash pulmonary edema. Workup included a left heart catheterization, which showed normal coronaries, and a CT angiogram, which revealed a large right renal mass. The patient was diagnosed with congestive heart failure and discharged after a 6-day admission on lisinopril, metoprolol, furosemide, and aspirin.

**Figure 1:**
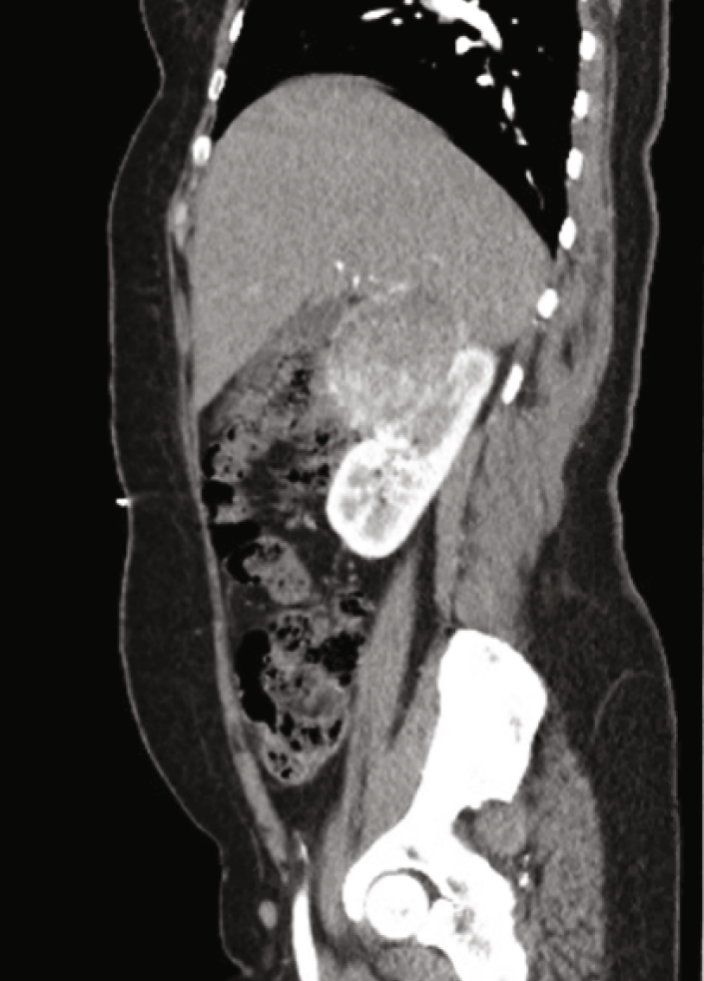
A 5.7 cm mass that appears to arise from the right kidney.

The patient was then referred to our center for robotic partial nephrectomy. In the office, the patient was well appearing and normotensive. An MRI renal mass protocol was ordered to further evaluate the renal lesion and revealed a 5.7 cm, heterogeneous, exophytic right upper pole renal mass (Figure 1). On the day of the surgery, the case began uneventfully; the robotic ports were placed and the abdomen was insufflated without any issues. The da Vinci Xi® robot was docked, but during the initial mobilization of the bowel, the patient developed severe hypertension up to 270/110 mmHg. Insufflation was immediately discontinued, and subsequently, her systolic blood pressure dropped to the 180s. Anesthesia started clevidipine and esmolol drips, and her blood pressure normalized to 110/80. The case was aborted, and the patient was admitted for workup of a possible pheochromocytoma.

Endocrinology was consulted and performed a complete evaluation. Significant findings included elevated plasma normetanephrine and metanephrine levels, which were 4533 pg/ml (normal 0-145 pg/mL) and 1410 (normal 0-62 pg/mL), respectively. She was diagnosed with a pheochromocytoma and started on phenoxybenzamine 10 mg twice a day and nifedipine 60 mg. Metoprolol was continued and lisinopril was stopped. She was discharged home.

**Figure 2:**
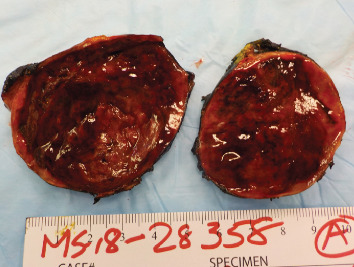
Final gross pathology specimen.

One month later, she returned to the operating room for robotic right adrenalectomy and partial nephrectomy. Initially, the medial attachments and vasculature feeding the tumor were controlled using clips and electrocautery. Next, the main adrenal vein was controlled and the mass was circumferentially dissected. The mass appeared to penetrate into the upper pole of the kidney, and the upper pole renal parenchyma had to be excised in order to resect the entire tumor. The renal parenchymal bed was then repaired using a barbed suture. The procedure was uneventful and the patient was discharged on postoperative day one. Final pathology revealed an 8.3 cm pheochromocytoma involving the adrenal gland and adjacent to the renal parenchyma (Figure 2).

## 3. Discussion

**Figure 3:**
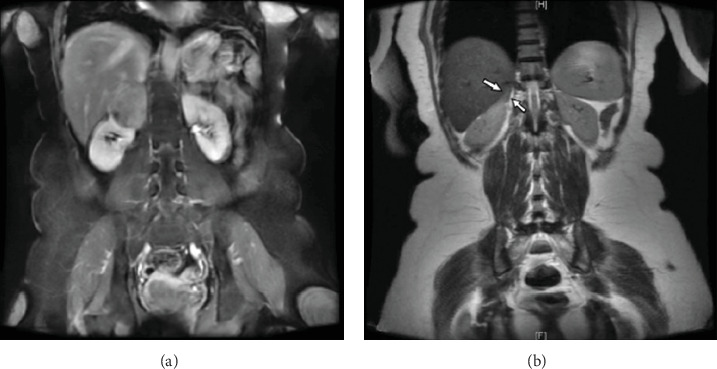
(a) The renal parenchyma can be seen creeping alongside the mass, a finding known as the “claw” sign, and is consistent with a mass of renal origin. (b) The right adrenal gland was identified on MRI and noted to be distinct from the renal mass.

In general, upper pole renal masses and adrenal masses can be distinguished by cross-sectional imaging. However, with large masses, it can be more difficult to identify boundaries between the kidney and adrenal gland. There have been a limited number of case reports in which adrenal masses were thought to be renal masses or vice versa [2–4]. In the case of our patient, the appearance of the mass on imaging was suggestive of a renal origin. First, MRI demonstrated the characteristic “claw” sign, which describes when the normal parenchyma of the organ of origin wraps around the developing mass (Figure 3(a)). Second, the adrenal gland was identified on imaging and thought to be displaced by the renal mass (Figure 3(b)).

Of note, one interesting point is that the patient was started on a beta blocker for the initial diagnosis of congestive heart failure. With her true diagnosis of pheochromocytoma, we would expect rebound hypertension. Singular beta blockade with the unopposed vasoconstriction leads to rebound hypertension; alpha blockade is more critical during the medical management of pheochromocytomas [5].

## 4. Conclusion

Current guidelines state that all incidentally diagnosed adrenal masses should undergo a metabolic workup to exclude pheochromocytoma [1]. However, no guidelines exist on the indications for metabolic workup when the mass is not clearly adrenal in origin. Considering that the incidence of pheochromocytomas is 0.2-0.6% among all patients with a new diagnosis of hypertension [1], we suspect that among patients with hypertension and a large upper pole renal mass the incidence of pheochromocytomas is much higher. Therefore, we recommend screening patients with hypertension and large upper pole masses for pheochromocytomas in order to better direct preoperative management.

## References

[1] Lenders J. W. M., Duh Q. Y., Eisenhofer G. (2014). Pheochromocytoma and paraganglioma: an endocrine society clinical practice guideline. *The Journal of Clinical Endocrinology and Metabolism*.

[2] Moslemi M. K., Al-Mousawi S., Dehghani Firoozabadi M. H. (2010). Renal cell carcinoma mimicking adrenal tumor. *Journal of Oncology*.

[3] Laroia S. T., Jain V., Rastogi A. (2015). Beyond the boundaries: enigma of distinguishing exophytic upper pole renal cell carcinoma from an adrenal mass. *World Journal of Nephrology and Urology*.

[4] Adas M., Koc B., Adas G., Yalcin O., Celik S., Kemik Ö. (2015). Pitfalls in the diagnosis of pheochromocytoma: a case series and review of the literature. *Journal of Epidemiological Research*.

[5] Darr R., Lenders J. W. M., Hofbauer L. C., Naumann B., Bornstein S. R., Eisenhofer G. (2012). Pheochromocytoma - update on disease management. *Therapeutic Advances in Endocrinology and Metabolism*.

